# Determinants of emigration and their impact on survival during dispersal in fox and jackal populations

**DOI:** 10.1038/srep24021

**Published:** 2016-04-06

**Authors:** Dror Kapota, Amit Dolev, Gilad Bino, Dotan Yosha, Amichai Guter, Roni King, David Saltz

**Affiliations:** 1 Mitrani Department of Desert Ecology, Jacob Blaustein Institutes for Desert Research, Ben-Gurion University of the Negev, Israel; 2 Science Division, Nature and Parks Authority, Jerusalem, Israel; 3 Centre for Ecosystem Science, School of Biological Earth and Environmental Sciences, University of New South Wales, Australia; 4 Society for protection of the nature, Tel Aviv, Israel

## Abstract

Animals disperse in response to poor resource conditions as a strategy of escaping harsh competition and stress, but may also disperse under good resource conditions, as these provide better chances of surviving dispersal and gaining fitness benefits such as avoiding kin competition and inbreeding. Individual traits should mediate the effect of resources, yielding a complex condition-dependent dispersal response. We investigated how experimental food reductions in a food-rich environment around poultry-growing villages interact with individual-traits (age, gender, body-mass) in two sympatric canids, red foxes and golden jackals, to jointly affect emigration propensity and survival during dispersal. Sub-adult foxes emigrated more frequently from the food-rich habitat than from the pristine, food-limited habitat, while adult foxes showed the opposite trend. During dispersal, adults exhibited lower survival while sub-adults did not experience additional mortality costs. Although fox mortality rates increased in response to food reduction, dispersal remained unchanged, while jackals showed strong dispersal response in two of the three repetitions. Jackal survival under food reduction was lowest for the dispersing individuals. While resources are an important dispersal determinant, different age classes and species experience the same resource environment differently and consequently have different motivations, yielding different dispersal responses and consequences.

What drives an animal to leave its natal-area or home range and disperse is a fundamental question, given the costs associated with such an action^1^. Theory discusses two main dispersal drivers: (1) escaping poor or deteriorated habitat/resource conditions^2^,^3^ and (2) avoiding kin competition and inbreeding^2^,^4^,^5^,^6^. While these drivers of dispersal are not mutually exclusive, they should manifest themselves differently in terms of the conditions under which dispersal occurs and the attributes of the dispersing individuals.

Whether to disperse or not under poor resource conditions is a complex decision: conditions may vary in space (favoring dispersal) or improve over time (favoring philopatry). Poor conditions affect the resident individual, but also the dispersing individual that will leave its natal area with lower energy reserves and deteriorated body condition, and thus have substantially lower chances of surviving dispersal and resettling^7^. Individuals having lower body-mass and competitive ability should be the most prone to leave a poor habitat, but should also experience the highest dispersal costs. This uncertainty in costs and benefits, possibly mediated by external and internal cues^7^,^8^, can reverse the decision to disperse in some cases. On the other hand, if dispersal is done not as an immediate response to stress, but for increasing ultimate benefits such as avoiding kin competition and inbreeding, it should be favored under particularly good resource conditions which allow the highest survival and resettling chances^9^,^10^,^11^. Dispersers of this type should be individuals with higher body-mass, and higher competitive and movement abilities. High dispersal rates are therefore predicted in the two extreme ends of the range of resource conditions^4^, but whether such dispersal response appears in a particular system depends on the specific context. The decision making process leading to dispersal should evolve based on its underlying cost-benefit balance, therefore gaining a broader view on why individuals disperse or not under particular circumstances requires examining not only the causes of dispersal but its consequences as well.

In this work we studied how resource conditions are combined with individual traits to affect emigration probability and the probability of surviving dispersal in populations of red foxes (*Vulpes vulpes*) and golden jackals (*Canis aureus*) inhabiting the same area in northern Israel. The populations of both species occupy two distinct habitats: a rich habitat resulting from poor sanitation in and around poultry farm villages, and a relatively poor habitat in the more pristine areas away from the villages^12^. Detecting effect of resource conditions on dispersal requires changing the per-capita resource level, i.e. changing the ratio between total resource level and the population density. Since the rich habitat in our study system supported a much denser population, no substantial differences may exist in the per-capita food intake between the habitats, given that populations in both habitats are stable over time^12^, and therefore differences in densities reflect steady-state differences in carrying-capacity. Changing per-capita food level can be achieved by artificially manipulating densities or resources^13^. In the rich habitat therefore, three abrupt food reduction experiments were performed, and under these conditions per-capita food levels dropped substantially^14^. On this basis, our general predictions were that populations occupying the two habitats would exhibit similar emigration rates, while experimental reductions of food would cause short-term emigration bouts. Survival probabilities during dispersal were predicted to be lower than survival at philopatry or after re-establishment, as the lack of a home range and continuous movement in an unfamiliar landscape are associated with risks of starvation and aggressive interactions^1^. Under experimental food reduction, dispersers should benefit, in terms of survival, from escaping poor conditions, but pay higher dispersal costs if the effect of poor conditions carries-over to the dispersal phase. Differences in survival between dispersers and non-dispersers should reflect this cost-benefit balance. To understand how individual traits (age class, gender and body-mass) mediate the effects of resources on dispersal behavior, we examined how individual traits interact with resource-related factors to affect emigration rates, and how these traits interact with dispersal to affect survival.

## Results

### Emigration

Of the 157 foxes and 39 jackals collared and tracked, we documented 28 dispersal events of 25 individual foxes, and 10 events of 10 individual jackals. The fate of 22 dispersal events of foxes and 8 of jackals could be determined, while the remaining events ended in disappearance during dispersal (but after emigration was conclusively documented). Of the known-fate dispersers, 8 foxes and 2 jackals successfully established a new home range (36% and 25%, respectively); all did so in the pristine habitat (i.e. their new home range did not cover any villages or poultry related facilities).

**Figure 1:**
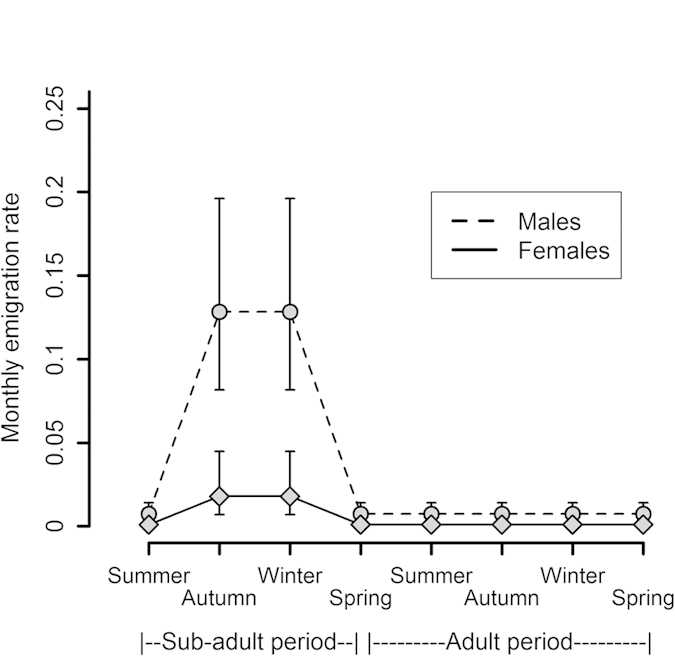
Model-averaged monthly emigration probabilities of foxes depending on age and gender. The time line follows sub-adults from their first summer and on (foxes become adults from their first spring and on), and plots are given separately for males and females. Bars represent 95% confidence intervals. Sub-adults emigrated only during autumn and winter, not during their first summer. Adults had the same basal emigration probability throughout the year. Males had higher emigration probability than females at all ages.

**Figure 2:**
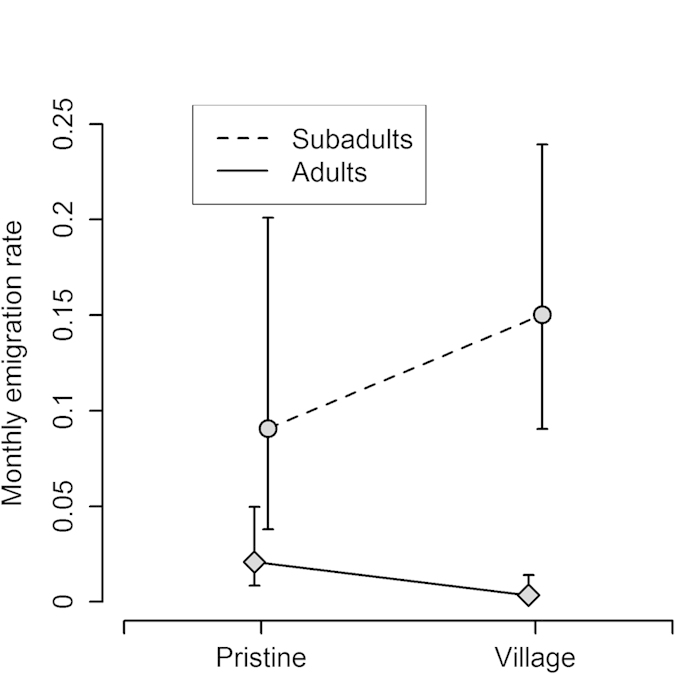
Monthly emigration probabilities of foxes are affected by the interaction between age class and habitat. Bars represent 95% confidence intervals. Sub-adult foxes emigrated more than adults in general. In addition sub-adults emigrated more from the village habitat than from the pristine habitat while adult foxes showed the opposite trend.

In the fox population, sub-adults dispersed more than adults, and males dispersed more than females in both age-classes (Fig. 1). Sub-adults began dispersing during autumn and winter (September-February): a model in which sub-adults also showed high emigration probabilities during their first summer was far less supported (Table 1 - model 14 versus 18). The two age classes differed also in relation to the habitat: adults emigrated more frequently from the pristine habitat, while sub-adults emigrated substantially more from the village habitat (Fig. 2). The three food reduction experiments did not prompt emigration in the fox population (Table 1 – model 2 versus 7).

**Figure 3:**
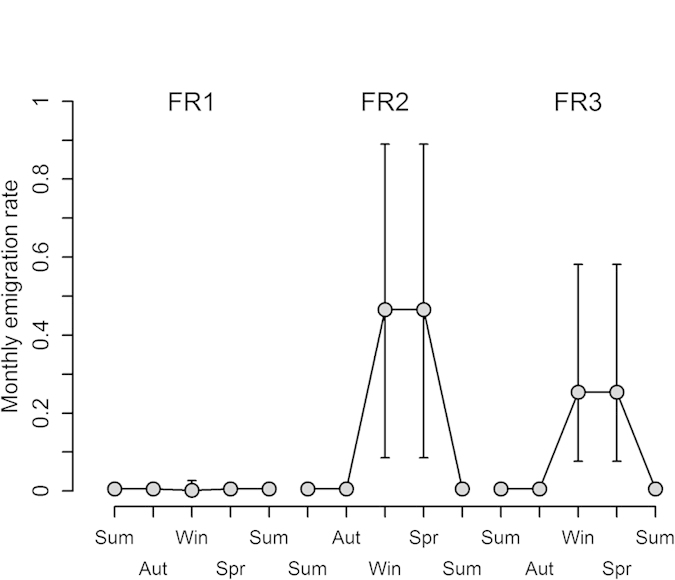
Model-averaged monthly emigration probabilities of jackals during three food reduction experiments (FR1, FR2 and FR3). Bars represent 95% confidence intervals. Emigration increased substantially following food reduction compared to the basal rate in FR2 and FR3. The large confidence intervals are due to the small sample size in each experiment, but nonetheless, the effect is strongly supported statistically. Estimations are weight-averaged over ages and genders.

In the jackal population, there is also evidence of differences in dispersal rates between age-classes, with sub-adults emigrating more frequently than adults (emigration probabilities of 0.97 and 0.995 for sub-adults and adults respectively). The prominent factor however is food reduction: two of the three FR experiments initiated strong emigration bouts among jackals (Table 2, FR2 and FR3 in Fig. 3), which started 2–4 months after the FR was initiated, and lasted about 3 months. The FR effect is strongly supported statistically (Table 2) despite the small sample sizes: in FR2, 3 of 6 jackals emigrated, and in FR3, 3 out of 5 emigrated. The limited number of individuals that were subjected to the FR experiments did not allow evaluating interactions between FR and the individual covariates, but qualitatively, emigrants during FR included adults and sub-adults, males and females, all in similar numbers.

### Survival during dispersal

**Figure 4:**
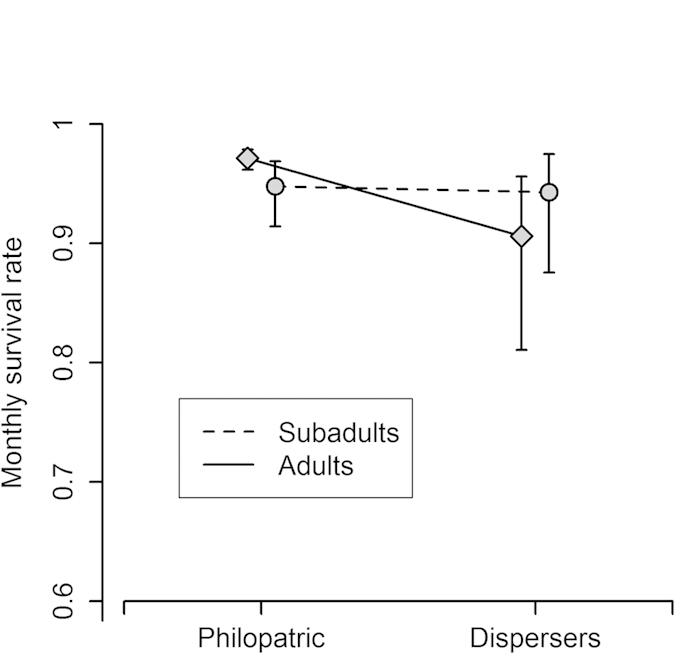
Monthly survival probabilities of foxes are affected by the interaction between dispersal and age-class. Dispersing adults suffer a substantial dispersal cost while Dispersing sub-adults suffer minimal or no cost.

For foxes, survival probabilities during dispersal were substantially lower than survival probabilities of philopatric individuals, or of dispersers after resettling (Table 3 and Supplementary Table S1). A model in which survival decreases permanently after emigration, namely also after resettling, was less supported (Table 3 - model 11 versus 12). The estimations imply that 66% of the dispersers are expected to successfully survive a six-month dispersal period (the maximum observed), while 80% of philopatric individuals should survive a period of the same length. Age plays a role in survival during dispersal: adults had lower survival during dispersal while survival of sub-adults was much less affected (Fig. 4). Two additional factors which affected survival in foxes and are important to consider when interpreting dispersal behavior are (1) habitat – foxes living around rural villages showed a higher survival probability than foxes living in the pristine habitat and (2) food reduction – fox survival was substantially lower under FR (Supplementary Table S1).

In the jackal population, there was evidence that both food reduction and dispersal had a negative effect on survival. The strength of this evidence is weaker than the rest of our results; however the model that includes both factors holds 0.29 of the probability, and the effect size of dispersal is large. It is therefore reasonable that the weaker evidence stems from sample size limitations, and not from redundancy of dispersal as a factor. The lowest monthly survival probability was therefore estimated for dispersing individuals that emigrated from FR-treated sites (the model-averaging estimate is 0.8969, the additive model estimate is 0.867 while the interactive model assigning a unique parameter for dispersers under FR, provides a slightly lower estimate of 0.862). Jackal survival also appeared to show an increasing trend over the years (‘Time’ factor in Table 4), and this factor was included here for controlling this source of variation.

## Discussion

Our experimental approach concentrated on dispersal under deteriorating conditions, yet the spectrum of factors we examined may reflect a wider range of conditions and dispersal behaviors. We therefore adopt a general perspective accounting for the two main dispersal drivers mentioned earlier (i.e. higher dispersal rates at the two extreme ends of the range of habitat conditions^4^) when interpreting and discussing our results.

While dispersal rates of sub-adult foxes were high, it does not appear to be the default - many sub-adults established their home range in their natal area (as opposed to, for example, yellow-bellied marmots (*Marmota flaviventris*) where nearly 100% natal dispersal was observed^16^). Thus, sub-adult fox dispersal is not entirely governed by outbreeding needs. Rather, the higher dispersal rate from the crowded village habitat indicates that dispersal is driven in part by limited available space (and consequently food) these sub-adults face after leaving their parents’ home range^12^, driving territorial antagonism and a higher frequency of male dispersal^2^,^17^.

Adult foxes, in contrast to sub-adults, dispersed more from the pristine habitat than from the village. Adults possess a well-defined home range and their dispersal behavior should therefore reflect mainly changes in local food availability. Since the higher population density in the village habitat should counteract the higher food abundance, per-capita food availability at steady-state is expected to be similar between the two habitats, and hence we did not expect a substantial difference in emigration rates. However, the higher home-range fidelity exhibited by adult foxes in the village suggests that they actually experienced either higher per-capita food levels and/or less variance in space and time. Comparing additional fitness components between the habitats supports this suggestion: foxes’ survival, body-mass, and the number of cubs per female are all slightly higher in the village habitat^12^,^14^. We suggest that the reason why population growth in the village habitat did not fully balance the per-capita food level is that under such high food density conditions, territorial behavior, rather than food limitations, controlled population growth.

The age × habitat interaction therefore suggests that the two age classes experience different types of resource limitation: sub-adults are driven to disperse by space (home range) limitations while adults are driven mostly by within-home range food limitations. Resource limitation is often approximated using population density. Using density as a predictor for dispersal in this case would suggest positive density-dependent dispersal in sub-adults, negative density dependence in adults and no density dependence overall. Studies correlating dispersal with density over distinct points in time or space show no consistency in their findings^3^,^15^,^16^,^18^. The studies that did not detect density dependence may have actually measured steady-state variation in density that is due to variation in habitat quality over time or space, such that no differences in per-capita resources exist^3^ or may have missed an important individual covariate (such as age class was in our study). Studying the effect of resource limitation by comparing dispersal across different populations should therefore consider not only variation in population density but also in habitat quality, and how per-capita resource availability is mediated by the individual state and traits.

Artificially manipulating densities or resources in a specific population perturbs the population’s steady-state and temporarily changes the per-capita resource level in a predictable manner, and therefore provides a reliable means of identifying dispersal response to changes in resource conditions. A review of studies that manipulated densities^3^ indeed found all of them to induce positive density dependent dispersal. Only one study known to us manipulated food in a natural population^13^. This study applied food supplementation to juvenile northern goshawks (*Accipiter gentilis atricapillus*) and found that supplemented birds dispersed less. Our food reduction experiments effectively reduced per-capita food levels as evident by the reduction in survival rates of both foxes and jackals; nevertheless, their effect on dispersal was mixed. Foxes did not disperse at all under food reduction. Jackals on the other hand showed a very strong dispersal response, and this is the only factor other than age that was found to affect emigration in this species. Yet, we documented this response only in two of the three repetitions of the experiment. Abrupt decrease in food levels therefore drives dispersal in some cases, but not in others. This variation between and within species suggests that stress-driven dispersal is by itself context- and condition-dependent. If the cost-benefit balance of such dispersal action varies according to the form of spatiotemporal variation and the individual’s state, it should evolve as a flexible strategy in which individuals integrate external and internal cues in making their decision whether to disperse or not^7^,^8^. Our results therefore provide evidence that poor resource conditions drive dispersal, but also indicate the complexity of these relationships.

Survival costs of dispersal in our system clearly exist. Such costs were documented previously^19^,^20^,^21^,^22^, but no attempt was made to study how these costs vary according to external and internal circumstances. In foxes, adults suffer a high survival cost during dispersal, while sub-adults experience little or no survival cost. Groups which experience high dispersal benefits and are selected for increased dispersal, are expected also to evolve physical, physiological or behavioral adaptations which facilitate dispersal and minimize its costs^23^. The differential cost among age-classes may hint to the existence of such hidden phenotypic adaptations in sub-adult foxes, and fits well with the exploration-exploitation paradigm^24^. Survival costs of foxes appear only during the transience phase, and do not continue after resettlement; the hypothesis of deferred dispersal costs^1^ is therefore not supported by our data. Under food reduction, dispersing jackals experienced lower survival than non-dispersers; therefore experiencing poor resource conditions prior to emigration imposes additional costs on the disperser, presumably via a carryover effect. The lack of support for a FR × Dispersal interaction apparently indicates no substantial ‘escaping’ benefit. However if all of the individuals remained at their home site, competition may have been much more intense, resulting in much lower survival rates, therefore the real escaping benefit may be much larger.

We have in our system two groups showing particularly high dispersal rates: sub-adult male foxes in the village habitat and jackals in the village habitat under food reduction. In both groups dispersal is driven by resource limitation, but its consequences for survival are rather different. The latter group clearly represents a case of stress-driven dispersal, where poor conditions both drive dispersal and impose additional survival costs on the disperser. The former group however seems to represent a mixture of circumstances. Individuals are driven to disperse by poor future prospects of space and food at the natal site, but at the same time experience low survival costs, presumably owing to good body condition. These circumstances may drive other parallel dispersal motivations such as avoiding kin competition and inbreeding. The effect of resource availability on emigration propensity and dispersal consequences is therefore mediated by individual state and traits. This presumably leads different individuals within the same resource environment to experience different circumstances, develop different motivations, and hence to exhibit different dispersal behaviors, making dispersal a complex, context- and condition-dependent phenomenon.

## Methods

Methods were carried out in accordance with approved guidelines. Specifically, all experimental protocols, including animal capturing, collaring and treatment were approved by the Israel Nature and National Parks Protection Authority (permission No. 38562).

### Study species, study area, and data collection

Red foxes and golden jackals are close species in terms of habitat and food requirements, demographic features, and movement behavior. The two species differ in size, with jackals having a mass ca. twice that of foxes (4.5 kg and 10.35 kg respectively for the studied populations) and are apparently competitively superior^25^. Foxes and jackals differ also in their social behavior – foxes are solitary while jackals tend to be in small groups^12^,^26^.

The study was conducted in the upper Galilee, northern Israel, over the years 2002 to 2010, in an area covering 9000 ha. The area is a Mediterranean mountainous region, where natural and agricultural lands are interspersed with agricultural villages that rely mainly on poultry farming. The fox and jackal populations in this region can be divided into two types – those inhabiting and foraging in natural and agricultural habitats (termed here ‘pristine’ habitats) and those relying intensively on human waste, particularly poultry carcasses, in and around rural villages^12^. Populations around rural villages are overabundant: fox densities were estimated at 16.1–23.3 individuals per km^2^ compared to 1.7–4.9 individuals per km^2^ in natural and agricultural areas, and similar trends appeared in jackal capture densities and transect counts^12^. In both habitats, capture densities and transect counts were constant over the years of study, suggesting that the populations are stable over time^12^.

Animals were captured during summer and autumn using soft foot-traps (Victory, ‘fox’ size), weighed, aged and gendered, and fitted with radio-collars containing a mortality sensor (Advance Telemetry Systems) weighing 120 gr for foxes and 190 gr for jackals. Sub-adults (individuals born in the spring before they were trapped) were easily distinguishable by body-mass and appearance, and we considered them sub-adults until the end of their first winter when they become sexually mature. In total, 157 red foxes and 39 golden jackals were radio-tracked on a weekly basis. Dispersed individuals were relocated by wide ranging vehicle and aerial searches.

Food reduction (FR) experiments were conducted in three villages by disposing poultry carcasses into scavenger-proof garbage bins which were provided to the farmers. This treatment effectively reduced food availability for the local fox and jackal populations^14^. Experiments were run for 4 months (January-April) in each village, and in three different years: 2006, 2007 and 2009 (one village each year). Cans were weighed and emptied once a week to a central animal-proof garbage bin. The mean total mass of carcasses disposed over the whole experimental period was 1.76 tonnes per village.

### Data analysis

Dispersal was defined as any permanent long-range movement out of a well-defined home range for adults, or out of the capture area for sub-adults. A long-range movement was defined as a movement of 2000 m or more, which is twice the home range mean diameter. Temporary long-range forays (several days or weeks) and permanent short-range movements (<2000 m) were not considered dispersal. Analyses were performed using the known-fate module in program MARK 6 27, that was originally designed for fitting generalized linear models to binary survival data by maximizing binomial likelihood functions. Here, both survival and fidelity probabilities (probabilities not to emigrate) were modeled as functions of time-related events such as FR and post-recruitment period, and individual covariates (species, gender, habitat, age and body-mass at capture).

In the fidelity analysis, individual tracking histories were clumped into 3-month intervals in which emigration events were encoded instead of mortality events, but unlike mortality, an individual’s record continued after the emigration event if it survived the dispersal and re-established a home range. Applying time-related effects to the relevant individuals was done by grouping individuals into trapping-cohorts according to year and site of capture. Practically therefore, fidelity probabilities (*F*) for each cohort (*c*) and time interval (*t*) were modeled as *logit*(*F*_*ct*_) = **X**_*ct*_**β**, where **X**_*ct*_ is the *ct* row of the design matrix, and **β** is the vector of model parameters. For instance, modeling lower fidelity of sub-adults during the first autumn and winter after recruitment, while accounting also for differences between genders, could be done by modeling only the fidelity probabilities of the first autumn and winter of each cohort as *logit*(*F*) = *β*_*0*_ + *β*_1*_Gender_+_*β*_2*_Age. Similarly, lower fidelity during FR was modeled by adding a parameter to fidelity probabilities of the cohorts participating in each of the experiments, during the experimental period.

Survival analysis was done using the same approach, but with survival probability as the dependent variable. The survival analyses presented here are concise and focus on the influence of dispersal on survival. Comprehensive survival analysis for foxes is reported in^14^ and for jackals in^28^. Here we included as explanatory variables only dispersal itself, factors that may interact with dispersal in affecting survival (age, gender, habitat and body mass) and factors that substantially contribute to the variance in survival and therefore must be accounted for when examining the factors of interest. We excluded factors that were examined in the two comprehensive analyses^14^,^28^ and found to have no clear contribution to variation in survival. Modeling dispersal as an independent variable affecting survival could not be done by simply grouping individuals to ‘non-dispersers’ and ‘dispersers’ because individuals emigrated and changed their status at different points in time. Dispersal as an independent variable was therefore modeled for each individual as a series of individual covariates, one for each time interval, holding its current status. We used two sets of such covariates. In one set a disperser status was assigned to the first three time intervals after emigration (dispersal events in this study lasted no longer than 9 months), while in a second set a disperser status was assigned to all time intervals after emigration. This way we could examine whether survival during dispersal decreases permanently or increases again once the animal re-settles.

Trapping-and-marking seasons extended over four months during which sub-adults still grew. We accounted for variation in body mass that is due to variation in capturing date by fitting body-growth curves, and using the residuals as the independent variable in the dispersal analysis (see Supplementary methods for further details). Fitting gender- and habitat-specific body-growth curves allowed also eliminating the correlation between these factors and body-mass.

Alternative models for emigration and survival probabilities were compared using Akaike Information Criterion (AICc) and ranked according to their strength of evidence^29^,^30^. Maximum likelihood estimations of the parameters were obtained by model averaging. Emigration probabilities reported in the results are 1 - *F*. For comparative reasons, both emigration and survival are translated and reported as monthly probabilities instead of 3-month probabilities.

Since jackals were less abundant than foxes in the study area, fewer jackals were captured and collared, and consequently sample size limitations were more prominent in this species. We attempted to perform equal analyses for both species; however the sample size limitations have led us to exclude in the jackal’s analyses some of the variables and potential interactions.

## Additional Information

**How to cite this article**: Kapota, D. *et al*. Determinants of emigration and their impact on survival during dispersal in fox and jackal populations. *Sci. Rep*. **6**, 24021; doi: 10.1038/srep24021 (2016).

## Supplementary Material

Supplementary Information

## Figures and Tables

**Table 1:** Models of emigration probability of foxes as dependent on habitat-related factors and individual covariates, ranked according to their AICc value.

	Model	AICc	ΔAICc	AICc Weights	Num. Par	Model Likelihood	Dev.
1.	{Habitat + Gender + Age + Habitat*Age}	167.992	0	0.458	5	1	157.90
2.	{Gender + Age}	170.402	2.410	0.137	3	0.299	164.36
3.	{Gender + Age + Gender*Age}	171.653	3.661	0.073	4	0.160	163.59
4.	{Habitat + Gender + Age + Habitat*Age + Habitat*Gender + Habitat*Age*Gender}	171.885	3.893	0.065	7	0.142	157.72
5.	{Habitat + Gender + Age}	172.322	4.330	0.052	4	0.114	164.26
6.	{Gender + Age + Body mass}	172.409	4.417	0.050	4	0.109	164.35
7.	{Gender + Age + FR}	172.425	4.433	0.05	4	0.109	164.36
8.	{Gender + Age + FR*Site}	173.109	5.117	0.035	6	0.077	160.99
9.	{Gender + Age + Body mass + Age*Body mass + Gender*Body mass}	174.019	6.026	0.022	6	0.049	161.89
10.	{Habitat + Gender + Age + Habitat*Gender}	174.173	6.181	0.020	5	0.045	164.08
11.	{Habitat + Gender + Age + Body mass}	174.336	6.344	0.019	5	0.041	164.25
12.	{Gender + Age + Body mass + Age*Body mass + Gender*Body mass + Age*Gender*Body mass}	175.995	8.003	0.008	7	0.018	161.83
13.	{Gender + Age + Body mass + Age*Body mass + Gender*Body mass + FR*Site}	176.962	8.970	0.005	9	0.011	158.70
14.	{Age}	190.824	22.832	0	2	0	186.80
15.	{Age + Body mass}	192.077	24.085	0	3	0	186.04
16.	{Habitat + Age}	192.184	24.192	0	3	0	186.15
17.	{Age + Body mass + Age*Body mass}	192.569	24.577	0	4	0	184.51
18.	{Age^†^}	203.720	35.727	0	2	0	199.70
19.	{Gender}	217.062	49.069	0	2	0	213.04
20.	{Habitat + Gender}	218.692	50.699	0	3	0	212.65
21.	{Gender + Body mass}	219.034	51.042	0	3	0	213.00
22.	{Gender + Body mass + Gender*Body mass}	220.108	52.116	0	4	0	212.05
23.	{Season}	228.951	60.959	0	4	0	220.89
24.	{Null}	237.534	69.541	0	1	0	235.52
25.	{Body mass}	238.628	70.635	0	2	0	234.61
26.	{Habitat}	238.882	70.889	0	2	0	234.86
27.	{Habitat + Body mass}	239.918	71.925	0	3	0	233.88
28.	{Habitat + Body mass + Habitat*Body mass}	241.417	73.425	0	4	0	233.36

In general ‘*’ denotes an interaction between two variables. Age refers to age-related effect during autumn-winter, while Age^†^ refers to age-related effect during summer-winter. FR refers to food reduction, and ‘site’ denotes the three FR sites. AICc weights are interpreted as model probabilities, therefore models 1–2 that together hold 59.5% of the weight, are more probable than all other models. These models contain age-related effect and gender, and the first model contains also an age-habitat interaction. Removing gender or age will result in a substantial decrease of the model probability (e.g. removing age effect: model 2 versus 19), while removing the age-habitat interaction results in smaller but still considerable decrease in probability.

**Table 2:** Models of emigration probability of jackals as dependent on habitat-related factors and individual covariates, ranked according to their AICc value.

	Model	AICc	ΔAICc	AICc Weights	Num. Par	Model Likelihood	Dev.
1.	{Age + FR*Site}	59.88	0	0.88699	4	1	51.62
2.	{FR*Site}	64.41	4.53	0.09194	4	0.1037	56.15
3.	{FR}	67.64	7.76	0.01831	2	0.0206	63.56
4.	{Season}	73.29	13.41	0.00109	4	0.0012	65.03
5.	{Age}	75.37	15.49	0.00038	2	0.0004	71.30
6.	{Gender + Age}	76.46	16.58	0.00022	3	0.0002	70.30
7.	{Null}	76.79	16.91	0.00019	1	0.0002	74.77
8.	{Age + Body mass}	77.31	17.43	0.00015	3	0.0002	71.15
9.	{Habitat + Age}	77.43	17.55	0.00014	3	0.0002	71.28
10.	{Gender}	77.57	17.69	0.00013	2	0.0001	73.49
11.	{Gender + Age + Body mass}	78.18	18.30	0.00009	4	0.0001	69.92
12.	{Body mass}	78.34	18.45	0.00009	2	0.0001	74.26
13.	{Body mass + Body mass}	78.75	18.87	0.00007	3	0.0001	72.60
14.	{Habitat}	78.79	18.91	0.00007	2	0.0001	74.72
15.	{Habitat + Gender + Age}	79.22	19.34	0.00006	4	0.0001	70.96
16.	{Habitat + Gender}	79.64	19.76	0.00005	3	0.0001	73.49
17.	{Habitat + Body mass}	80.38	20.50	0.00003	3	0	74.22
18.	{Habitat + Gender + Age + Body mass}	80.86	20.97	0.00002	5	0	70.47

In general ‘*’ denotes an interaction between two variables. FR refers to food reduction, and ‘site’ denotes the three FR sites. AICc weights are model probabilities, therefore models 1–3 that together hold 99.7% of the weight, are far more probable than all other models. These models contain two factors: FR effect and age-related effect. Removing any of the two will result in a substantial decrease of the model probability (e.g. removing FR effect: model 1 versus 5).

**Table 3:** Models of survival probabilities of foxes as functions of dispersal, habitat-related effects and individual covariates, ranked according to their AICc value.

	Model	AICc	ΔAICc	AICc Weights	Num. Par	Model Likelihood	Dev.
1.	{Dispersal + FR + Habitat + Age + Dispersal*Age}	503.108	0.000	0.293	8	1	486.89
2.	{Dispersal + FR + Habitat}	503.778	0.670	0.209	6	0.7153	491.65
3.	{Dispersal + FR + Habitat + Age}	504.045	0.937	0.183	7	0.6259	489.88
4.	{Dispersal + FR + Habitat + Body mass}	505.699	2.591	0.080	7	0.2737	491.53
5.	{Dispersal + FR + Habitat + Dispersal*Habitat}	505.706	2.598	0.080	7	0.2728	491.54
6.	{Dispersal + FR + Habitat + Gender}	505.800	2.693	0.076	7	0.2602	491.63
7.	{Dispersal + FR + Habitat + Body mass + Dispersal*Body mass}	507.709	4.602	0.029	8	0.1002	491.50
8.	{Dispersal + FR + Habitat + Gender + Dispersal*Gender}	507.719	4.611	0.029	8	0.0997	491.51
9.	{Dispersal + FR}	508.792	5.685	0.017	5	0.0583	498.70
10.	{(FR}	511.952	8.845	0.004	4	0.012	503.89
11.	{Dispersal}	519.479	16.371	0.000	2	0.0003	515.46
12.	{Dispersal permanent}	521.883	18.775	0.000	2	0.0001	517.86
13.	{Null}	522.477	19.369	0.000	1	0.0001	520.47

In general, ‘*’ denotes interaction between two variables.FR refers to the effect of food reduction, “Dispersal permanent” to permanent post-emigration (reduction in survival also after establishment) effect and “Dispersal” to temporary post-emigration effect (reduction in survival only during dispersal). The two first models, holding 68.5% of the weight, contain temporary dispersal effect, habitat and FR effects, while the most probable model contains also an interaction between dispersal and age.

**Table 4:** Models of survival probabilities of jackals as functions of dispersal and food reduction, ranked according to their AICc value.

	Model	AICc	ΔAICc	AICc Weights	Num. Par	Model Likelihood	Dev.
1.	{Time + FR}	150.690	0.000	0.384	3	1	144.55
2.	{Time + FR + Dispersal}	151.239	0.549	0.292	4	0.76	143.01
3.	{Time + FR*Dispersal}	153.337	2.646	0.102	5	0.76	143.01
4.	{FR}	153.964	3.274	0.075	2	0.26	143.00
5.	{Time + Dispersal}	154.521	3.831	0.057	3	0.26	143.00
6.	{Null}	154.860	4.170	0.048	1	0.19	149.89
7.	{Time}	155.064	4.374	0.043	2	0.14	148.38

In general, ‘*’ denotes interaction between two variables. FR refers to the effect of food reduction. The three first models, holding 77.8% of the weight, contain the FR effect, dispersal effect and a time trend over the years. The support for dispersal as an explanatory variable exists but is weaker than for foxes, because of sample size limitations.
