# Supplementary material for: Determinants of emigration and their impact on survival during dispersal in fox and jackal populations
Source: Sci Rep. 2016 Apr 6;6:24021. doi: 10.1038/srep24021 (PMC4822138; doi:10.1038/srep24021)
Supplement: Supplementary Information [file srep24021-s1.pdf]

Determinants of emigration and their impact on survival during dispersal in fox and jackal populations

A. Dror Kapota, B. Amit Dolev, C. Gilad Bino, D. Dotan Yosha, E. Amichai Guter, F. Roni King, G. David Saltz

Supplementary Table S1: Maximum likelihood estimates for monthly survival probabilities of foxes and jackals. Since none of the foxes dispersed under food reduction no estimations are available for this category.

	**Foxes**		**Jackals**	
	**Normal**	**Food reduction**	**Normal**	**Food reduction**
**Philopatric**	0.9750496	0.9129498	0.9726426	0.9224002
	0.9635699-0.9829757	0.8402393-0.954365	0.9373479-0.988302	0.8319437-0.96615
**Dispersal**	0.9381598		0.9620556	0.8969211
	0.8696964-0.971817		0.8915453-0.987374	0.7528937-0.961315

Supplementary method: Sub-adult body-mass Standardization

When examining the effect of body-mass on the propensity to disperse, we must have accounted for the variation in body-mass between sub-adult individuals that is due to differences in capturing times during their growth period. The way to do so is estimating a body-mass growth curve for the mean individual, and using the residuals from this curve instead of raw body-mass values. These residuals reflect the individual variation around the mean without the temporal variation due to different capturing times during the growth period.

First, adult body-mass was regressed against habitat and gender, both were statistically supported, and their coefficients were estimated. Sub-adult body-mass was then modeled as a logistic growth over time: $= \frac{bm_{max}}{1+\frac{(bm_{max}-bm_{min})}{bm_{min}}e^{-rt}}$. A set of logistic equations varying according to the linear dependency of bm_{max}, bm_{min} and r on habitat and gender was fitted, and coefficients for bm_{min} and r were estimated. Coefficients for bm_{max} were taken from the adult body-mass model and integrated into the logistic equations prior to the fitting process. The most probable model in the set (model with lowest AICc) suggested that bm_{min} depends only on habitat and r is constant over both habitat and gender groups. This model was used for calculating residuals as described above.
